# Female Physicians and Female Rights

**Published:** 1872-02-15

**Authors:** J. O. Herriott

**Affiliations:** Valparaiso, Ind.


### FEMALE PHYSICIANS AND FEMALE RIGHTS.

BY J. O. HERRIOTT, A.M., M.D., VALPARAISO, IND.

On reading the reported discussion of the female physician question, by the American Medical Association, assembled in San Francisco, California, May 2d, 1871, we are prompted to express our humble views on this so-called "vexatious question."

That spirit of disobedience which ruled in the breasts of our first parents, and for which both were cursed, is still rife, and manifests itself in ways innumerable. But now, as in the beginning, woman takes the lead in violating the Divine commands, and breaking those seals which were written and stamped upon her by the hand of the Creator. Any attempt she may make to avoid that curse pronounced upon her as a penalty for disobedience, by transgressing those laws written upon her organization, as precepts for her guidance in conduct and duty, will assuredly bring upon her additional trouble and sorrow.

There are points in the organization of the sexes so nearly alike as to indicate that many actions and duties of life may be performed by either one or the other. Such are the organs of prehension and locomotion; but there are points in the organization of woman which limit her sphere of conduct and action. If no duties separate from those which belong to man had been intended for woman, then she would have taken the full form and organization of man: the type of the one would have been the exact type of the other. But they are different mentally, morally, and physically; and therefore a different class of duties is predicated for them.

This diversity of adaptation of means to an end pervades every department of nature, although our finite minds are not in all cases permitted to perceive it; but in the case of woman the record is so plain "that he that runneth may read." And this principle of nature is recognized and acted upon everywhere by the artisan and mechanic. He who builds a house chooses from the different kingdoms of nature that which best suits his purpose. He knows that wood will not serve the use of stone, nor stone of lead, nor lead of iron or steel; but that each have a sphere of usefulness according to their physical properties; and, if adjusted in the fabric accordingly, they suit the purpose for which they were chosen. But if improperly placed, the fabric would be comparatively useless. And the same principle is manifest and recognized in the case of man and woman. The one cannot take the place of the other in the social fabric, without defeating the great object and end for which they were created and designed. As well might we expect the sun and the moon, having departed from their apparent circuit in the heavens, to produce the regular routine of "day and night, winter and summer, seed time and harvest," as to expect man and woman, having departed from their appropriate spheres in the social system, violating the laws of adaptation and the precepts of nature, to produce order and harmony in the social relations of life.

As respects woman's equality, which she complains is not recognized, every sensible man will grant her full equality with himself. And why should he not? She is a segment of the social circle, without which it cannot be complete. If this circle cannot be complete without both of these, how can we estimate a superiority of one above that of the other? The idea of superiority is absurd.

Let us turn our attention, then, to the more practical part of this subject, and see how this new order of things, which these women would inaugurate, will conform to natural law. By mutual consent, let the present order of things be reversed. Let the females turn into the offices—medical and legal, to the workshops, and to the fields, and let the males turn into the kitchens, the laundries, parlors, etc., there to manage all the affairs of the household, including the care of new-born infants.

But perhaps she may say she does not intend to have any of these latter incumbrances. What then? Has she conceived some new method by which the race is to be perpetuated and preserved, in which she will take no part or lot? a plan of which neither philosophers, physiologists, nor even the Creator himself has dreamed? Let woman reflect that she, and she only, was created and expressly adapted to meet this emergency; and meet it she must, or suffer the penalty of violated natural law. Any effort on her part to evade it will be a vain effort to revolutionize the government of nature, and defeat the design for which she was created. There are times and seasons in the life of woman, when the activity of her peculiar functions will incapacitate her from attending to professional duties, especially if she has a proper regard for her health. What is to become of these duties in the meantime? Can she arrange with her patients to call only when it may suit her health and convenience? This is obviously impracticable as regards the practice of medicine. Or does she propose to abrogate those functions which may interfere with the even tenor of her way? This would be an attempt to alter the designs of the Creator concerning her, and a sad reflection on His wisdom and goodness in making her a woman.

Why she should desire to forsake her own sphere, and seek pursuits incompatible with her nature and organization, is very strange indeed, and looks very like fault-finding and ingratitude towards her Maker. It can only be accounted for by the false estimate she makes of her own dignity and importance in society. She must either undervalue her own qualifications, as being inadequate to fill the place designed for her, or that it is inadequate for the exercise of all the gifts bestowed on her.

It is often asked, What is woman's sphere? In answer to this, we reply, that to specify every act which woman may do or not do, would be an almost endless task. Her organs of prehension and locomotion give great latitude for her conduct, but there are peculiarities in her organization which limit her actions and duty to the domestic circle. This is the star by which she is to be guided in the proper direction of duty and conduct. Her mission as wife and mother are specific: the perfectly helpless and dependent condition of the period of infancy is alone sufficient to limit her conduct and duty to this circle. She is its "guardian angel," and to engage in any pursuit which might interfere with the duties she owes to it, will be detrimental to her own best interests, the interests of her household, and that of society. And this circle is amply spacious for the exercise of all those gifts and graces which the Creator has bestowed upon her. The modeling and giving tone to society, which has its origin in this circle, give ample scope to signalize herself and give her notoriety. Many, very many, great men in this and other countries, have attributed their greatness and success to the early training of their mothers. This alone without a thousand other considerations, ought to be sufficient to assure her of the honor and dignity of her station, and to reconcile her to it.

Is the practice of medicine, and some other pursuits, to which many women of the present day are aspiring, compatible with the dictates of nature, and calculated to elevate her in the scale of importance, and make her more honored, loved and respected? No. The hopes she entertains on this subject are false hopes, based on false premises. Water will not run up hill. Would an urgent summons at the hour of midnight, in a rain or snow storm, to attend the sick, be compatible with her own health and safety during the activity of the function of menstruation? Would a summons, while she herself is in a state of gestation, to attend a sister at parturition, be compatible with the safety of her own life and health, and the normal development of her fœtus? Is the period of lactation, a period specially dedicated by nature for the exercise of natural affection, a proper time for her to attend to the professional wants of others, and thus repudiate and crush that sacred principle, by neglecting the wants of her own household, and the fruit of her womb? Common sense will answer to all these questions, emphatically, No! Would the life of a soldier, a sailor, a midshipman, a surgeon or a general, and many other pursuits which we could point out, and which require much bodily as well as mental vigor, be appropriate pursuits for her beautiful and delicate organization? And above all, would they be compatible with that modesty which is the crowning glory of her sex? We leave the answer to the instinctive feelings of every sensible man and woman.

If woman has a right to engage in any pursuit for which she is not by nature adapted, she has a right to all the pursuits which belong to man. But the moment she assumes this responsibility, she will perceive a gradual alienation of the heart and affections of man from her; and she will perceive, also, that she is regarded more as a convenience than an ornament or accomplishment of society. Soon man will neglect or refuse to pay those tributes of regard, courtesy, kindness, and protection, and he will plausibly and effectually excuse himself by saying that, as she has assumed the responsibility of being her own man, she has forfeited all claims upon his gallantry. Thus that delicacy and refinement of feeling which has always existed between the sexes in a highly cultivated state of society, and gives zest and charm to it, will gradually become obliterated.

Woman's lot in this life is a hard lot; but not more rugged and arduous than that of her brother, eating bread "in the sweat of his face," and waging war with the thorns and the thistles that grow up in the pathway of life, while the multiplying of "sorrow and conception, and bringing forth children in sorrow," are "the portion of her cup." These are the conditions of their lives, and each are equipped and adapted by nature to meet these conditions, but neither can lay down their armor, or exchange it for any other, and meet the struggle of life successfully.

There is no nation on the face of the earth where woman is more loved, honored, and protected, by law and common usage, than in our own country; and if woman is dissatisfied with this tender regard manifested for her, and is determined to urge this departure, and forfeit this protection, courtesy and kindness, she will interfere very much with her own happiness and comfort, and tarnish her dignity and honor. It will beget a coarse community of sentiment and conduct between the sexes repugnant to the feelings of every virtuous and refined mind.

In those countries where woman is forced from the sphere which the Creator designed for her, and made an abject slave, and man is "lording it over God's heritage," as among the North American Indians, and in India, there is nothing but degradation and misery of both sexes.

It is very gratifying to be able to say that it is not the best part of female society who sanction this departure, but only those who are ambitious of notoriety, but have not talents to signalize themselves in their own appropriate spheres. The most dignified part of female society reject with scorn and contempt the whole thing. And it gives us equal pleasure to be able to say that we have never known an instance where man has tried to signalize himself by acting the part of woman.

What we have said on this subject we consider sufficient to demonstrate that the dictates of nature, as exhibited in the organization of woman, is antagonistic to her practising medicine, and many other pursuits to which she aspires.

How, then, shall we treat these would-be female physicians? Shall we recognize them as co-laborers in the practice of medicine? We answer, emphatically, No! We cannot encourage them in any course which would do incalculable injury to themselves and to society at large.

Our mother Eve was made for man,
Though not to be his slave;
But helper and his solace be,
While traveling to the grave.

This view of the subject will, no doubt, be considered by some as ultra, and applicable only to the body physical, and not to the body social. We answer to this that the body physical and social are almost exact types of each other, and that the body social is as sensitive to abnormal departures from propriety and rectitude as the body physical is to departures from the standard of health. At first the cause of the departure from the standard of health may be so slight as scarcely to be seen or felt; yet, by sympathy of the other organs of the body, a tumult and turmoil may be raised sufficient not only to threaten, but destroy, life. And so it is with the body social: an error or evil may be introduced into society, at first apparently so slight and trivial as not to be seen, or its evil effects appreciated; yet, by its presence and affinity for other errors and evils, may raise a tumult and confusion sufficient not only to threaten but destroy the good order and harmony of the body social.

That females have a capacity to comprehend the science, and skill to practice the art of medicine, we have no doubt. But this has nothing to do with the question. The question at issue is, should a woman forsake her womanhood, and engage in pursuits which would interfere with the duties she owes to the domestic circle, and thus produce confusion in the social system?

Man has as much right to invade woman's territory as she has his; but the truth is no right exists on either side. For he is no better qualified to fill her place than she is to fill his, and according to the designs of nature the good order and harmony of society require each to do their own appropriate work.

The idea of females practising medicine being in accordance with the laws of her organization, and compatible with the duties she owes to her household and society, and calculated to promote her true interests and happiness, is a false idea, a false conception, and would have properly disposed of itself ere this time if left to its own tendencies. But since its inception, false modesty, false courtesy, and false philanthropy, on the part of medical men, have been the pabulum of its life and growth. Without these it never could have attained its present magnitude, which now calls for medical legislation.

Many medical men, without examining its true merits and incompatibility with the precepts of nature, have taken their position "on the rail," where they can easily change attitude without changing base; sometimes with both legs on one side "the rail," and sometimes on the other, as may best suit the complexion of popular sentiment. But when a doubt arises which side may prevail or be victorious, we find a leg on each side "the rail." This was the ridiculous position of some professed patriots during the time of the southern rebellion. In the latter case the rebellion was against the constitution and laws of the nation; in the former against the constitution of woman and the laws of nature.

Man's mind is weak at best estate,
But some there are who would be great;
But then, alas! we see how frail
They look when "sitting on the rail."

It is surprising to contemplate the different views which different men entertain on this subject. Some medical men in good standing in the profession,and some in high places, regard it as a normal movement, calculated to elevate woman in the scale of importance. This view necessarily implies that she is degraded and down-trodden. If this were really true, the appeal to elevate her would be just and proper; but to elevate her to positions for which she is not adapted and ordained by nature, reminds us very much of a method they have in these days, of elevating culprits above a crowd of spectators, with a rope round their necks—elevating them to death or ruin. Others view it as a dangerous kind of complaint which has crept into the body social, uncertain as to crisis, but threatening the life and health of the profession; and are therefore treating it with "placebos," the composition of which is largely "neutral mixture." Others, again, we are pleased to say, are treating it on its true merits, by saying to woman, "I can't do your work, and you can't do mine."

What we have said on this subject, we believe to be in strict accordance with the mental, moral, and physical teachings of the organization of woman, and in consonance with the best interests of society.

